# A case of gastrointestinal stromal tumor metastasized to the left ventricular myocardium

**DOI:** 10.1186/s40792-022-01433-6

**Published:** 2022-04-25

**Authors:** Tadashi Takasaki, Takashi Tsuji, Shogo Nakayama

**Affiliations:** Department of Cardiovascular Surgery, Japanese Red Cross Osaka Hospital, Osaka, Japan

**Keywords:** Heart tumor, Myocardial tumor, GIST, Gastrointestinal stromal tumor

## Abstract

**Background:**

Gastrointestinal stromal tumors (GISTs), which are the most common soft tissue tumors of the gastrointestinal tract, originate from Cajal interneurons. The main metastatic sites of GISTs are the liver and intra-abdominal cavity, and metastasis to the heart is rare.

**Case presentation:**

The patient was a 78-year-old man who was diagnosed with a rectal GIST 20 years previously. Since then, he had undergone repeated operations for metastasis. A follow-up thoracoabdominal computed tomography scan 4 months prior to the operation revealed GIST metastasis to the left ventricular myocardium. The patient wanted the tumor removed and consequently underwent an operation. The surgical findings showed a 3-cm × 3-cm mass in the lateral wall of the left ventricle. The mass was resected from the left ventricular wall in the shape of a tear drop. The left ventricular cavity was closed with a 4–0 polypropylene mattress suture and continuous suture. Postoperative histopathological findings showed nodular tumor growth consisting of bundles of spindle-shaped cells in the myocardium. The margins were negative. Immunostaining showed c-KIT (CD17) positivity and CD34 positivity, consistent with GIST metastasis.

**Conclusions:**

This case involved GIST metastasis to the heart muscle, which has rarely been reported worldwide.

## Background

Gastrointestinal stromal tumors (GISTs) are the most common soft tissue tumors of the gastrointestinal tract, originating from Cajal interneurons. Ninety percent of GISTs stain positive for KIT (or CD117), a receptor tyrosine kinase [1]. The main metastatic sites of GISTs are the liver and intra-abdominal cavity, and metastasis to the heart is rare [2]. Here, we report a case of rectal GIST that metastasized to the myocardium, and we discuss the relevant literature.

## Case presentations

The patient was a 78-year-old male.

*Chief complaint* Asymptomatic.

*History of present illness* The patient was diagnosed with rectal GIST 20 years ago and received an endoscopic resection. Five years ago, he underwent total anorectal partial mesolectal resection (TAMIS), open pancreaticoduodenectomy and splenectomy. Two years ago, he underwent transanal resection of recurrent rectal GIST. Three months ago, left ventricular myocardial metastasis was suspected by thoracoabdominal contrast-enhanced CT. Adjuvant imatinib was withdrawn due to the appearance of skin symptoms along with tearing and itching of the eyes. The patient was referred to our department for resection.

*Medical history* Rectal GIST, COVID-19, *Klebsiella pneumoniae*, transit ischemic attack.

*Allergy* Imatinib (skin symptoms, eye symptoms).

**Fig. 1:**
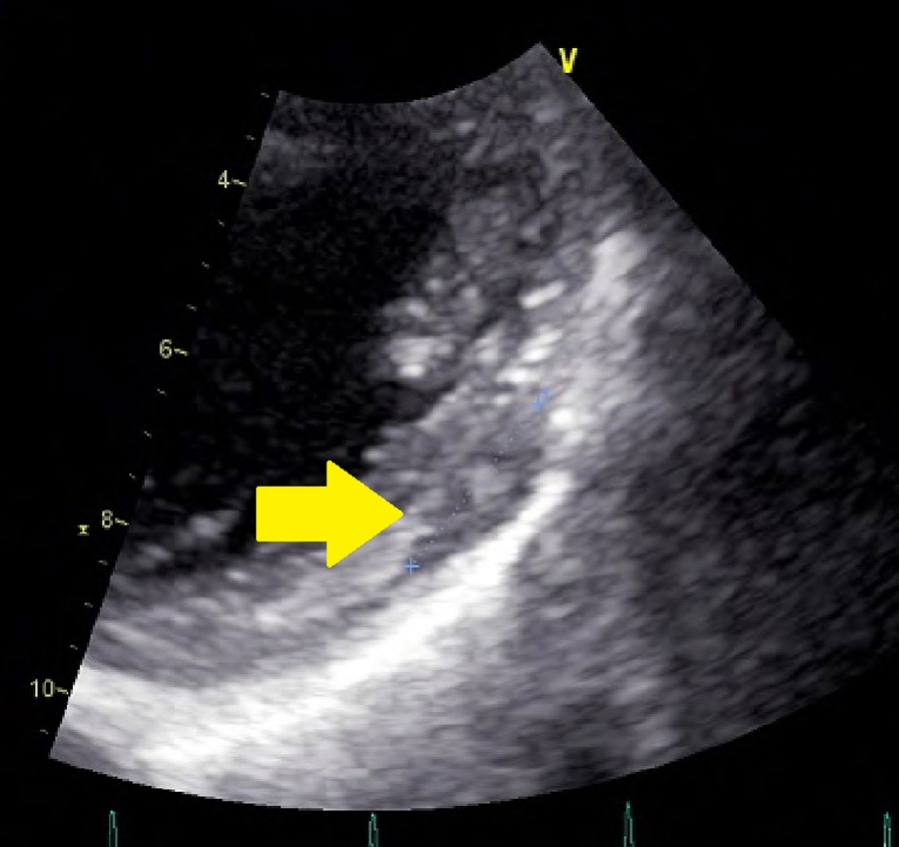
Cardiac echography showing a tumor with a 12-mm diameter in the wall of the left ventricle (arrow)

*Transthoracic echocardiogram* There was a 12-mm limbic, clear, low-echoic area on the lateral wall of the left ventricle (Fig. 1). The left ventricular end-diastolic diameter was 42 mm. The left ventricular end-systolic diameter was 29 mm. The left ventricular ejection fraction (modified Simpson) was 60%. The IVC was 15 mm. E/A was 0.90. E/E' was 10.3. The heart valves worked correctly. The left ventricular wall motion was normal.

**Fig. 2:**
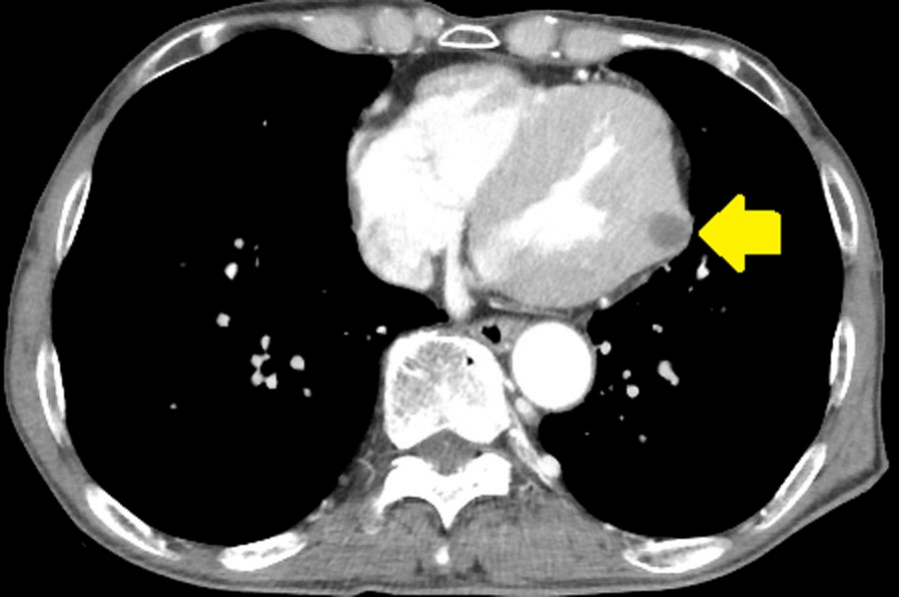
Computed tomography of the chest showing a defect (11 × 11 mm in diameter) in the left ventricle (arrow)

*CT* Left ventricular mass (11 × 11 mm) (Fig. 2).

**Fig. 3:**
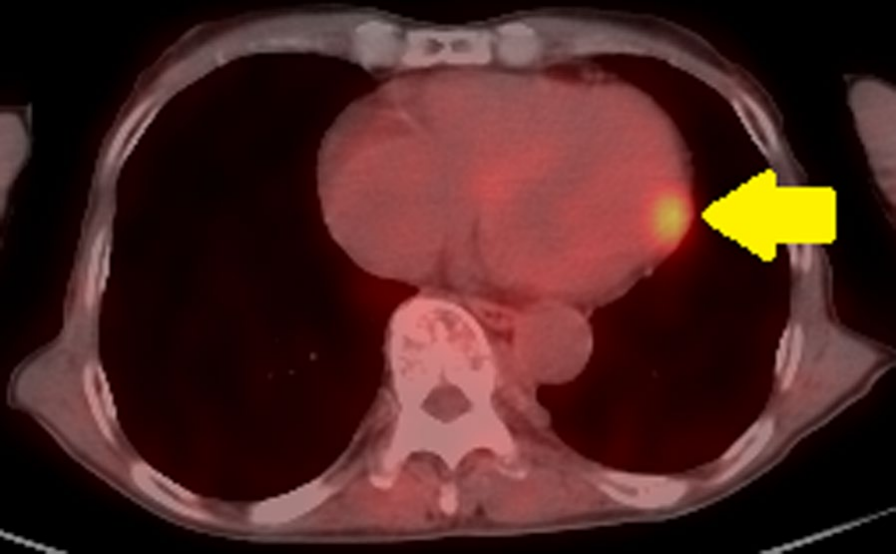
PET showed a high degree of FDG accumulation in the left ventricular myocardium (arrow)

*PET image* High degree of FDG accumulation in the left ventricular myocardium (Fig. 3).

**Fig. 4:**
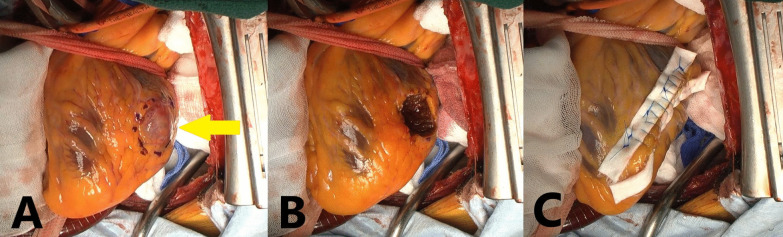
Intraoperative view of the gastrointestinal stromal tumor (GIST) before resection (yellow arrow) (**A**). Intraoperative view showing the heart after tumor resection (**B**) and the heart with closing sutures and felt strips (**C**)

*Operative findings* The patient was approached through a midline sternal incision, and extracorporeal circulation was established. The ascending aorta was clamped, and myocardial protection fluid was infused in both the antegrade and retrograde directions to arrest the heart. When the heart was arrested, a circular mass was observed at the end of the diagonal branch (Fig. 4A). It felt like hard rubber to the touch. The mass was trimmed in the shape of a tear drop and resected from the left ventricular wall with a sharp-edged knife (Fig. 4B). A part of the mass had extended into the left ventricular cavity. A 10-mm felt was placed around the resected hole, and the felt was threaded and closed with a 4–0 polypropylene horizontal mattress suture and continuous suture (Fig. 4C). The operation time was 2 h and 39 min, the total extracorporeal circulation time was 60 min, and the aortic clamp time was 32 min.

*Postoperative course* The pathological findings were negative for tissue fragments. The patient was discharged home on the 12th postoperative day without any complications. Postoperative transthoracic echocardiography showed that the left ventricular end-diastolic diameter (41 mm), left ventricular end-systolic diameter (29 mm) and left ventricular ejection fraction (60%) were not changed. The heart valves worked correctly. The wall motion of the left ventricle at the myectomy site was significantly (mildly to moderately) reduced, but the patient had no symptoms.

**Fig. 5:**
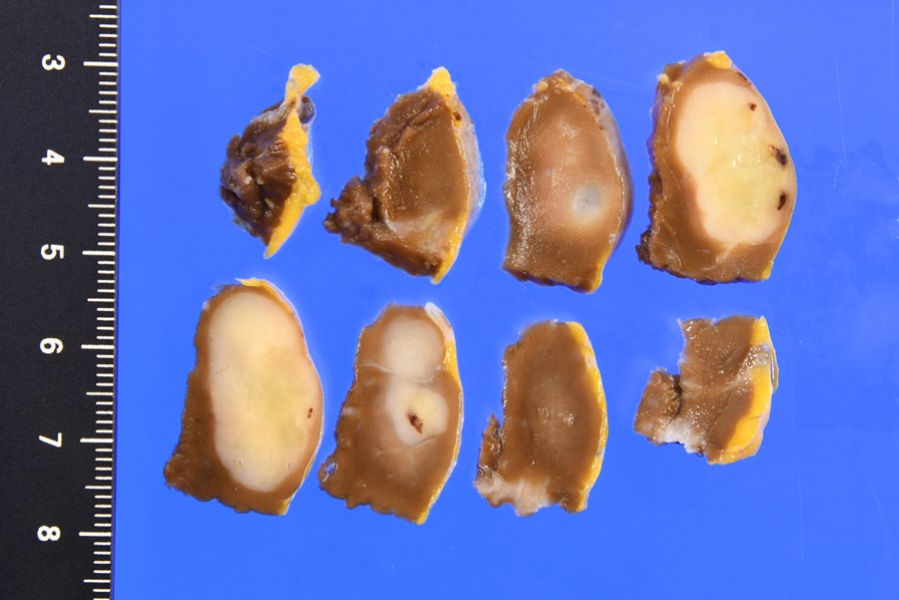
Macroscopic view of the resected specimen (cut surface) showing the tumor, which was 12 × 24 × 13 mm in size

*Excised specimen* The tumor was well defined, with some necrosis. The tumor diameter was 12 × 24 × 13 mm (Fig. 5).

**Fig. 6:**
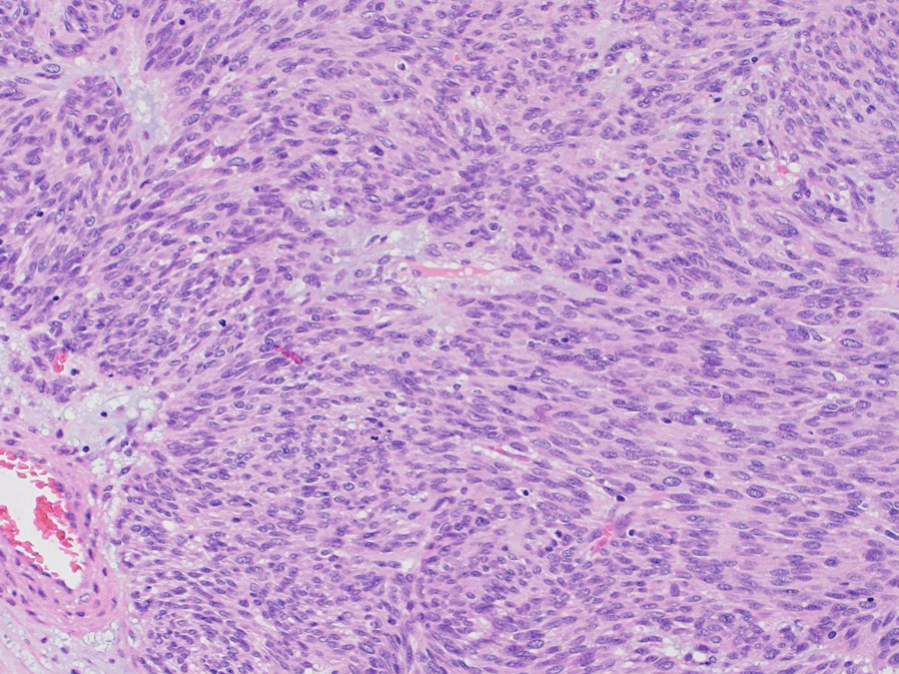
Hematoxylin and eosin staining (magnification ×100). Nodular tumor growth consisting of bundles of spindle-shaped cells is seen

**Fig. 7:**
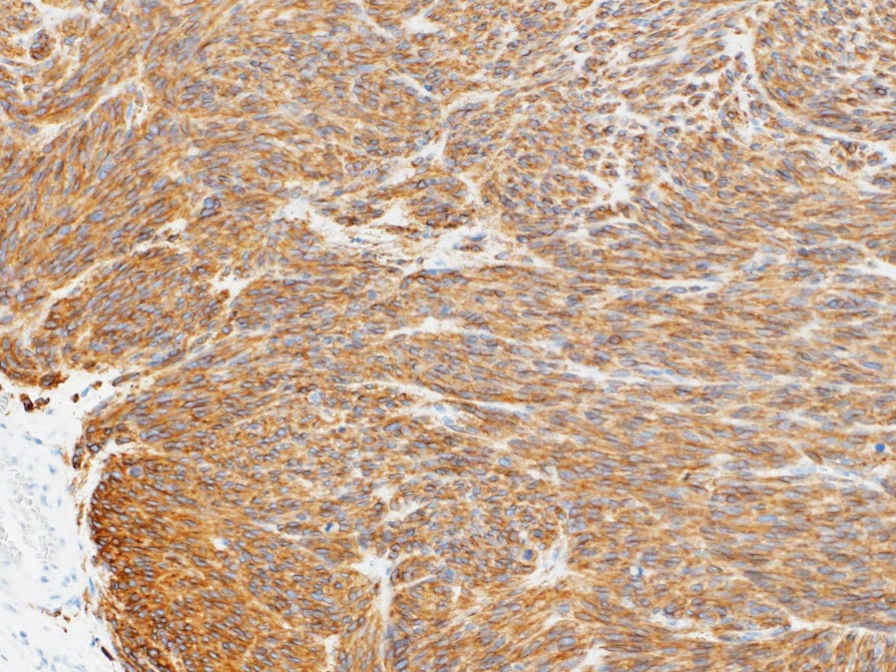
Positive immunostaining for c-KIT (CD117) (magnification ×100) indicated a gastrointestinal stromal tumor (GIST)

**Fig. 8:**
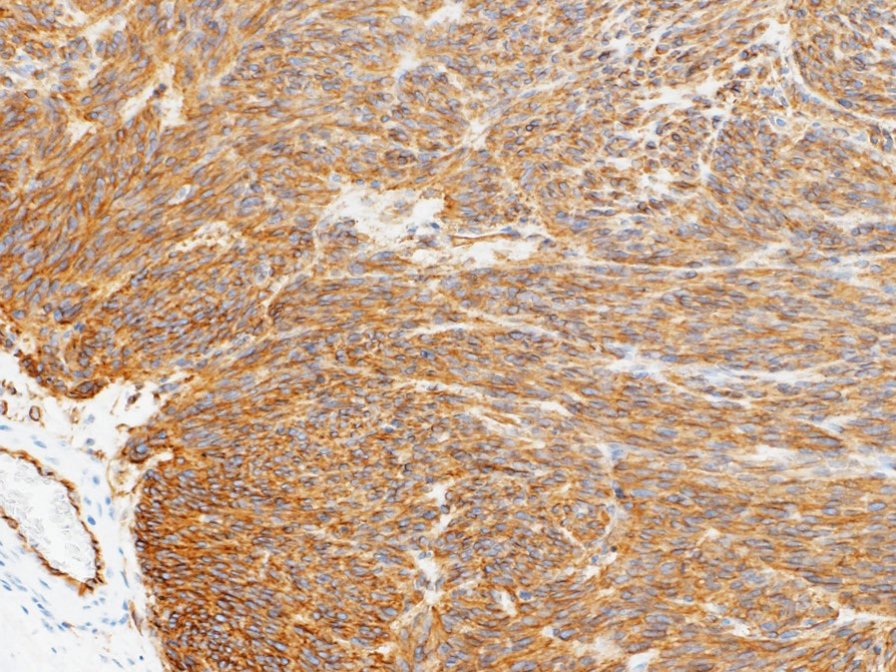
Positive immunostaining for CD34 (magnification ×100) indicated a gastrointestinal stromal tumor (GIST)

Histopathological examination revealed nodular tumor growth consisting of bundles of spindle-shaped cells in the myocardium (Fig. 6). Hyaline degeneration and focal hemorrhage were scattered in the tumor. The tumor showed an increased chromatin volume, but there was no significant cellular atypia or increased mitotic figures that would indicate sarcoma. On the specimen, the margins were negative. Immunostaining was positive for c-KIT (CD117) (Fig. 7) and CD34 (Fig. 8).

## Discussion

GISTs are mesenchymal tumors that exhibit morphologic and immunophenotypic features similar to those of Cajal interneurons, the pacemaker cells that regulate gastrointestinal stromal tumors. Approximately 90% of GISTs stain positive for KIT (or CD117), a receptor-type tyrosine kinase [1]. GISTs occur primarily in the stomach (60–70%), small intestine (20–25%), colon and rectum (5%), or esophagus (< 5%) [3]. GISTs most commonly metastasize to the liver and abdominal cavity [4]. Myocardial metastasis of GISTs is very rare. There have been two reports of metastasis to the left ventricular myocardium [2], one report of metastasis to the right ventricular myocardium [5], and one report of metastasis to the right atrium [6]. The immunostaining results of the cardiac tumor in this case were consistent with metastasis of a GIST, suggesting that the tumor had originated in the rectum and metastasized to the heart. Based on the number of cases reported thus far, this is a very rare case.

Imatinib is known to be effective in the treatment of GIST and is the first-line treatment for metastatic and recurrent GISTs. The results of clinical trials show that nearly 70% of patients have a partial response [7]. Imatinib was also started in this case, but its use was discontinued due to adverse skin reactions. Based on the size of the tumor measured by preoperative transthoracic echocardiography and chest CT, complete removal of the tumor was thought to be possible.

## Conclusions

We report a rare case of left ventricular myocardial metastasis of GIST.

## Data Availability

Not applicable.

## Abbreviations

GIST: Gastrointestinal stromal tumor
CT: Computed tomography
